# Physical Activity Habits and Determinants, Sedentary Behaviour and Lifestyle in University Students

**DOI:** 10.3390/ijerph17093272

**Published:** 2020-05-08

**Authors:** Aida Carballo-Fazanes, Javier Rico-Díaz, Roberto Barcala-Furelos, Ezequiel Rey, José E. Rodríguez-Fernández, Cristina Varela-Casal, Cristian Abelairas-Gómez

**Affiliations:** 1 Health Research Institute of Santiago de Compostela (IDIS), University Hospital of Santiago de Compostela-CHUS, 15706 Santiago de Compostela, Spain; aidacarballofaz@gmail.com (A.C.-F.); roberto.barcala@uvigo.es (R.B.-F.); 2 CLINURSID research group, Psychiatry, Radiology, Public Health, Nursing and Medicine Department, Universidade de Santiago de Compostela, 15782 Santiago de Compostela, Spain; 3 Faculty of Education Sciences, Universidade de Santiago de Compostela, 15782 Santiago de Compostela, Spain; javier.rico.diaz@usc.es (J.R.-D.); geno.rodriguez@usc.es (J.E.R.-F.); 4 REMOSS research group, Faculty of Education and Sport Sciences, University of Vigo, 36005 Pontevedra, Spain; zequirey@uvigo.es (E.R.); cristinavarelacasal@uvigo.es (C.V.-C.)

**Keywords:** physical exercise, sedentary life, causes, lifestyle, undergraduate students

## Abstract

University students, as a result of their lifestyles, represent a section of the population that is most likely to adopt sedentary behaviours. The aim of the present study was to analyse the determining factors dictating the performance of physical activity as well as sedentary behaviour among university students. A total of 608 students (64.6% women) from the University of Santiago de Compostela (Spain) were selected by stratified random sampling to take part in the study, which involved completing a questionnaire on lifestyle and physical activity. Of the participating students, 69.6% indicated that they performed physical activity; the main reasons given were to maintain fitness and for health, while a lack of time and laziness were the principal reasons given for abandoning or not taking up physical exercise. Significant associations were established between not doing physical activity and the time exposed to screens, time studying, feeling low and smoking; on the other hand, associations could be seen between doing physical activity and the participation of relatives (parents, mothers, partners, older siblings and friends) in physical activity, as well as a positive sense of satisfaction relating to physical education taught in schools. In conclusion, most of the university students did some physical activity, which was associated with less sedentary behaviour, while the influence of school physical education and of the habits of relatives played an important role.

## 1. Introduction

Sedentary behaviour includes those activities that imply an energy expense ≤ 1.5 METs, whilst physical inactivity is defined as an insufficient level of physical activity (PA) that does not meet current recommendations [1]. Both behaviours have increased in recent years due to changes in the physical, social and economic environment of our current society [2]. 

There are great physiological and psychological benefits of PA that have also been associated with health benefits [3,4]. Nevertheless, in spite of the World Health Organization’s (WHO) recommendation to perform moderate-to-vigorous PA [5], levels of sedentary living and physical inactivity are alarming [6].

Scientific evidence has shown that high levels of sedentary living and physical inactivity are related to an increased risk of obesity [2,7,8], different types of cancer [9,10,11,12], metabolic syndrome [13,14], cardiovascular diseases [12,15,16,17], diabetes [7,12,14,17] and mortality [17,18]. Furthermore, reduced PA [19] has also been associated with an increased risk of depression [20,21] and anxiety [22,23]. In the same way, an inverse relation has been demonstrated between sedentary behaviour and overall quality of life, happiness and perceived health [24,25].

University students, due to the time spent in classes, studying or in front of computers, form part of the population most at risk of adopting sedentary behaviour [26]. Different studies have shown that the transition to university studies is usually associated to a decrease in PA and an increase in sedentary behaviour [27,28] as a result of lifestyle changes, as well as psychosocial factors [29].

Considering the increase in sedentary behaviour levels and the association of this with physical inactivity in young people, as well as the related problems, it seems important to investigate whether associations could be found between PA and the lifestyle of university students. Therefore, the aim of the present study was to analyse the determining factors connected to university students´ PA and sedentary behaviour.

## 2. Materials and Methods 

### 2.1. Participants

Students from the University of Santiago de Compostela (Santiago Campus, Spain) took part in the research. They were selected by means of a process of random sampling stratified by groupings (qualifications and course) in order to guarantee the equal inclusion and significance of the whole Campus population. This population was made of 21.612 students (13.693 women) distributed across 20 centres. For the calculation of the sample size, a confidence interval of 95.5% and a sampling error of 4% were established. Also, a proportional allocation was carried out to account for gender. The final sample was set at 608 students (393 women, 64.6%). The participants belonged to different study disciplines (degree or master) from the area of Arts and Humanities, Sciences, Health Sciences, Social and Legal Sciences and Engineering and Architecture. The characteristics of the students are shown in Table 1. Participation was voluntary and verbal consent was obtained from all students. No formal consent or Ethical Committee assessment was required for this study since no personal data was collected from the participants. The study followed the ethical principles established by the Helsinki Convention. 

### 2.2. Tools

A validated questionnaire addressing the students´ PA and lifestyle was used [30]. This was adapted to the characteristics of the University of Santiago de Compostela and was modified according to the contributions of 10 experts, all graduates in either Sports Sciences or Psychology, using the Delphi method. 

The questionnaire was made up of 6 sections: (1) general information (age, sex, academic course, PA habits, duration of PA, reasons for taking or not taking exercise; (2) sedentary behaviour (learning time, screen time); (3) health problems (backache, feeling low, annoyed or nervous and sleep issues); (4) perception of health and physical condition; (5) drug consumption; (6) interpersonal relations (PA habits of parents, mothers, partners, friends and siblings); (7) physical education (PE) in school (enthusiasm for PE in school and participation in extra-curricular sports activities). 

### 2.3. Procedure

The questionnaires were completed by the students during classes, in the classrooms of the university department in which they were studying for bachelor´s or master’s degrees, under the supervision of one researcher. The participants had the entire time of the class to focus calmly on the questions. The same information was communicated to the students in order to avoid bias and to maintain the quality of data. 

### 2.4. Statistical Analysis

The results were analysed with the statistical software SPSS for Mac (version 25.0, Chicago, IL, USA). This consisted of a descriptive analysis of the categorical variables (expressed in absolute and relative frequencies), and the continuous variables (expressed in measurements of central tendency [median] and the dispersion [interquartile range]). To determine the normality of the sample the Kolmogorov-Smirnov test was applied. The Chi-Square test was used to analyse the association between the performance of PA (yes/no) and the rest of the categorical variables. As a means to study the inter-subject differences of the continuous variables Mann–Whitney’s U-test was applied. The significance value for the all the analyses was established at *p* < 0.05. 

## 3. Results

### 3.1. Demographics and Reasons behind the Performance and Abandonment of Physical Exercise 

Table 1 shows the demographic statistics of the sample. A total of 608 university students answered the questionnaire. About 70% of the sample confirmed their participation in PA, and most of the students (~90%) undertook PA during the week. Regarding gender, 74.0% of the men and 67.2% of women performed PA. Nevertheless, the only established association with gender as a variable was related to the number of minutes during which PA was undertaken, with the greater proportion of males indicating training sessions of over 1h (χ^2^_(2)_ = 54.554, *p* < 0.001). No association was found between the practice of PA and the different study disciplines of the participants.

Table 2 shows the students’ reasons for undertaking PA and the Table 3 the categorization of those reasons. A larger number of male students said they do PA for reasons like fun, to meet friends, because they like it or enjoy feeling physically competent. Nevertheless, the motive with greatest difference between men and women is competition; 81.1% of the men who indicated undertaking PA, do it for this reason, compared with just 39.0% of the women.

Regarding the motives for not doing PA, there were no differences between the men and women´s answers. The most popular reason was a lack of time (75.1%) followed by laziness (70.8%). Almost half of the students indicated that they did not undertake PA due to a lack of suitable facilities (49.2%) or a lack of training companions (49.1%). About a third of the sample declared that they did not feel competent (36.8 %) or that they did not enjoy exercise (33.5%). 

Ninety-seven participants confirmed they had given up doing PA in the past. Among the reasons for giving up, the inability to combine studies with PA stood out (89.7%), followed by the influence of friends´ abandonment (45.4%). Other reasons included the demands of trainers, beginning a new relationship or parental prohibition, selected by 21.6%, 31.6% and 18.6% respectively. Additionally, 27.8% indicated that they had given up PA having suffered some type of injury. There were no associations between abandonment motives and gender. 

### 3.2. Sedentary Behaviour: Screen Time and Time Studying

A record was taken of the number of hours per day that the participants spent studying [Monday to Friday: 2 (1–4); weekend: 2.5 (1–4)]; watching TV [Monday to Friday: 1 (0–2); weekend: 2 (1–3)] and using the computer/video games [ Monday to Friday: 2 (1–3); weekend: 2 (1–4)], and the results were compared between those who do PA and those that do not. Significant differences were found in the number of hours of study from Monday to Friday [PA Yes: 2 (1–4); PA Not: 3 (2–5); *p* = 0.008] and from Monday until Sunday [PA Yes: 2.4 (1.4–3.9); PA Not: 2.7 (1.7–4.7); *p* = 0.030]; also in the total hours of screen time (television + computer/video games) from Monday until Friday [PA Yes: 3 (2–4); PA Not: 3 (2–5); *p* = 0.045].

Table 4 shows the differences in the variables related to sedentary behaviour between men and women. It demonstrates how men differ from women regarding sedentary behaviour, with screen time dominating for male students, while female students dedicated more time to studying.

The variables in Table 4 show no significant differences between those men who did PA and those that did not. In the case of the women, those that did PA had lower learning time from Monday until Friday [PA Yes: 2 (2–4); PA Not: 3 (2–5); *p* = 0.01] as well as lower screen time from Monday until Friday [PA Yes: 2 (2.5–4); PA Not: 3 (2–5); *p* = 0.045].

### 3.3. Health Problems

Of the students, 27.3% admitted to regularly suffering backaches; 18.1% to feeling low; 25.0% to feeling irritable; 36.0% to feeling nervous; and 26.6% to having difficulty sleeping. The only variable with an association to undertaking PA was that of feeling low. Of those students who did PA, 15.4% confirmed feeling low. On the other hand, among the students who did not undertake PA, the equivalent figure was 24.3% (χ^2^_(1)_ = 6.970, *p* = 0.008).

Applying the same analysis according to gender, for the women there were no associations between the variables related to health and doing PA or not. In the case of the men, there was an association between doing PA and not feeling low (χ^2^_(1)_ = 4.800, *p* = 0.028). 

Nor was there any association between the health variables and the duration of sessions amongst those undertaking PA.

### 3.4. Perception of Health and Physical Condition

Associations were found between doing PA and self-perception of each individual´s physical condition, unlike the perception of health and diet. Of those students who perceived their own physical condition as bad, 54.3% undertook PA. These percentages increased among the students who perceived their condition as regular (69.8%) or good (80.2%; χ^2^_(2)_ = 24.640, *p* < 0.001).

Examining the gender variables, in the case of the female students, there is an association between perceived physical condition and the level of PA. Specifically, the percentages of women who did PA increased as the perceived physical condition variable rose in quality (bad: 51.6%; regular; 71.2%; good: 74.5%; χ^2^_(2)_ = 13.956; *p* = 0.001). The same pattern was observed in the case of men, not only in perceived physical condition variable (bad: 60.5%; regular; 66.7%; good: 87.8%; χ^2^_(2)_ = 13.507; *p* = 0.001), but also in the perceived health variable (bad: 16.7%; regular; 68.2%; good: 77.6%; χ^2^_(2)_ = 12.107; *p* = 0.002).

**Figure 1:**
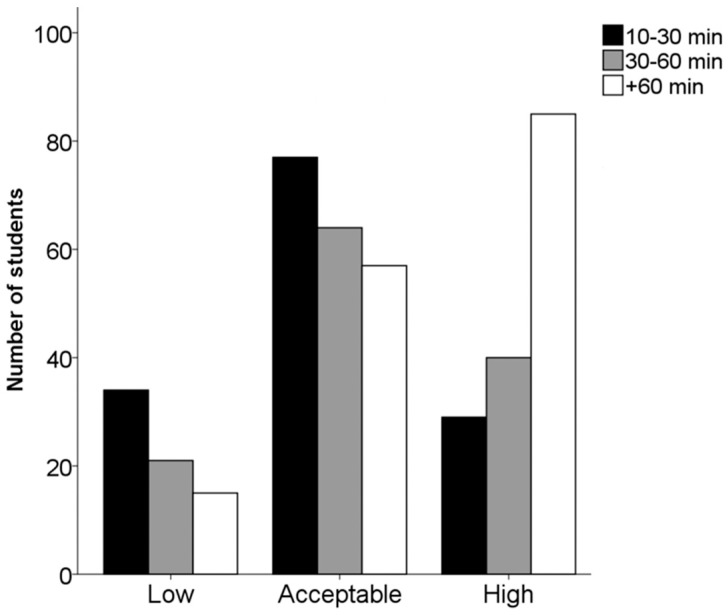
Relation between perceived health perception and duration of exercise sessions.

Associations were also found between perceived physical fitness and the duration of exercise sessions. Figure 1 shows a distinct tendency among those students who perceived their own physical condition as good, with a greater percentage of students undertaking sessions of more than one hour (χ^2^_(2)_ = 39.534; *p* < 0.001).

No associations were established between any of the variables and the choice of weekends or weekdays as the timetable for exercise. 

The pupils were also questioned about the number of meals or snacks that were taken per day. In general, most of the students ate less than 4 daily meals (61.4%). Out of this 61.4%, 65.1% did PA. On the other hand, out of the remaining 38.6% that confirmed taking 4 or 5 daily meals or snacks, the percentage of pupils that performed PA was 76.5% (χ^2^_(1)_ = 8.740; *p* = 0.003). Another association was between the number of meals and gender. While the women made up 59.5% of the students who took less than 4 daily meals, this increased to 73.1% of the students who reported 4 or 5 meals (χ^2^_(1)_ = 11.582; *p* = 0.001).

### 3.5. Drug Consumption

Doing PA and the consumption of tobacco were also associated. The students that did PA made up 60.7% of the smokers. Nevertheless, the percentage of students who confirmed regular exercise made up 71.7% of not smokers (χ^2^_(1)_ = 5.161; *p* = 0.023). No association was established between PA and the consumption of alcohol and other drugs.

Regarding gender, 70.0% of the men consumed alcohol every week, as opposed to 61.5% of the women (χ^2^_(1)_ = 4.487; *p* = 0.034). Another association was established between gender and the consumption of alcohol more than two days per week (men: 9.3%; women: 2.3%; χ^2^_(1)_ = 15.099; *p* < 0.001). As for the consumption of other drugs in the last 30 days, a greater proportion of men fell into this category with 19.5 %. (women: 9.7%; χ^2^_(1)_ = 11.838; *p* = 0.001).

### 3.6. Interpersonal Relations

**Figure 2:**
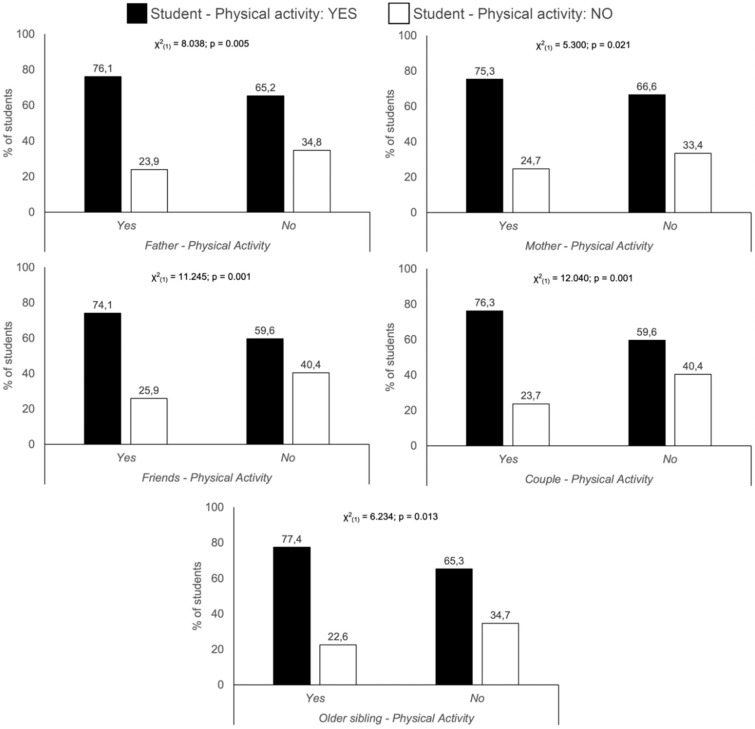
Relation between the students’ performance of physical activity and that of relatives.

Figure 2 shows that PA on the part of mothers, fathers, partners and older siblings of the students has a significant association with the habits of the students themselves. In each case, one can see the proportion of students that exercise if the particular relative (or friend or partner) also does PA. In addition, the connection was more positive when both father and mother undertook exercise (χ^2^_(1)_ = 14.692; *p* < 0.001. Regarding siblings, while Figure 2 shows the influence of an older sibling exercising, this association was not apparent with younger siblings. 

Regarding gender, the most positive association for the male students was with the PA of fathers, mothers, both parents, friends, and partners (χ^2^_(1)_ = 9.142, *p* = 0.002; χ^2^_(1)_ = 4.538, *p* = 0.033; χ^2^_(1)_ = 5.421, *p* = 0.020; χ^2^_(1)_ = 10.269, *p* = 0.001; χ^2^_(1)_ = 9.632, *p* = 0.002, respectively) whereas for the women the most influential factors were ordered as follows; both parents, partners and older siblings (χ^2^_(1)_ = 11.570; *p* = 0.001; χ^2^_(1)_ = 11.102, *p* = 0.001; χ^2^_(1)_ = 6.357, *p* = 0.012, respectively).

### 3.7. Physical Education in School and Extra-Curricular Physical Activity

Although the percentage of students that enjoyed PE in primary school and that continue to undertake PA (71.3%) is greater than the percentage of students that currently do PA despite not enjoying school PE (62.3%), there is no significant association (χ^2^_(1)_ = 3.764; *p* = 0.052). However, examining the case of the female students separately, the association is clear. Specifically, 70.0% of the women who enjoyed primary school PE continue to exercise at present; on the other hand, only 54.2% of those who did not enjoy primary school PE still exercise (χ^2^_(1)_ = 6.765; *p* = 0.009).

**Figure 3:**
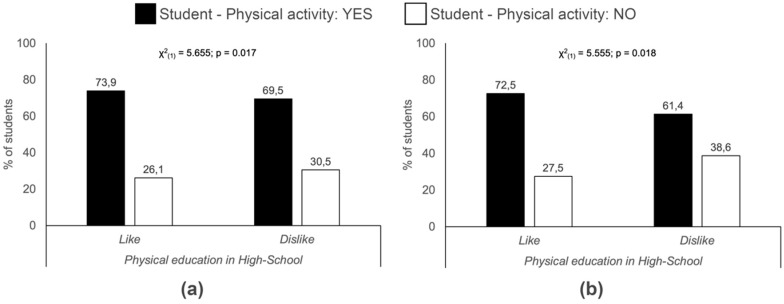
Relation between current physical activity and enjoyment of physical education in secondary school. (**a**) Total sample; (**b**) Women.

**Figure 4:**
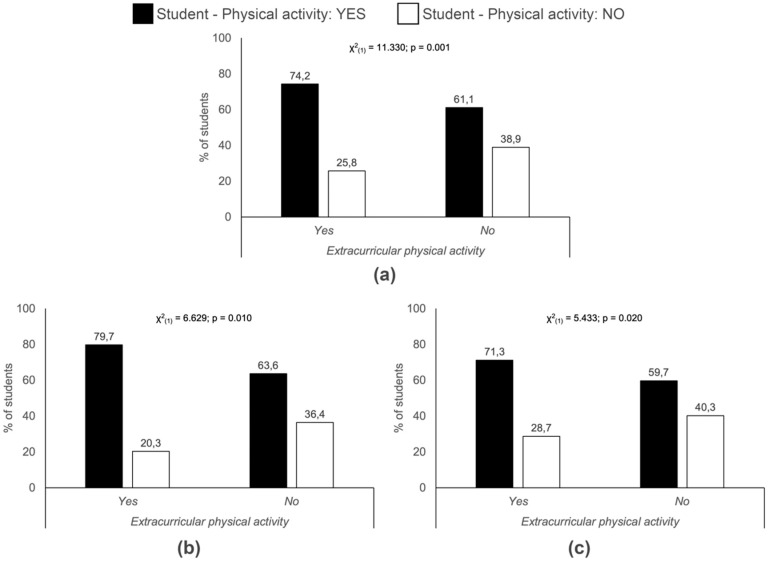
Relation between physical activity and participation in extra-curricular sports activities organized by the educational centre. (**a**) Total sample; (**b**) Men; (**c**) Women.

As regards the enjoyment of PE in secondary school and undertaking PA currently, there is an association both within the complete sample and the female students taken separately (Figure 3). With regard to extra-curricular PA organised by the education centre, an association was established for the total sample as well as the men and the women (Figure 4).

## 4. Discussion

The present study analysed some factors that determine levels of PA and sedentary behaviour amongst a sample of university students, to make visible their lifestyle, as well as any motivations and barriers which might encourage or discourage the undertaking of PA. The university population´s motivations to undertake PA deserve to be the object of scientific study for various reasons: these students make up a high proportion of young people as a whole [31]; some of them become future teachers or health professionals, taking on an educational role on within the wider population [32]; although doing PA is a habit that should be acquired at an early age, university living is a key period in the development of healthy habits that could influence adult life [33].

Our results show that approximately 70% of the polled university students were doing PA. Earlier studies obtained slightly higher numbers in young adults (78.5%) [34] and slightly lower in a group between 18 and 49 years (about 65–70%) [35]. Gender has appeared as a variable that is associated to PA, with men more predisposed to perform PA than women [36]. A study carried out with university students from 24 countries showed half of the women were physically inactive, as opposed to 25% of the men [37]. Our results showed a greater proportion of the women did not do PA, but the inter-gender differences were not significant. Nevertheless, there was a statistically significant difference in terms of exercise duration (over 1 hour), which was greater in men. The differences between men and women in their performance of PA might be influenced by the cultural patterns of the past, which encouraged sports more among men and boys [38].

Regarding the reasons for undertaking PA, 51.8% of the participants chose “maintain fitness” as a very important motive, followed by the reason “health”, considered very important by 43.0%. There were differences between men and women, the male students do PA for reasons like fun, to meet friends, to feel physically competent and especially for competitive reasons, in higher numbers than the female students. Similar differences for the genders were obtained in a previous study [39]. The lack of time, followed by laziness were the main motives for being physically inactive, without significant differences between men and women. Similarly, previous investigations indicate the lack of time as the main reason for physical inactivity among university students [39,40]. As for giving up PA, almost 90% of the students of our study who indicated this choice, gave the reason for the abandonment as an inability to combine PA with studies, re-stating the absence of time that was mentioned previously [41]. 

Our study considered sedentary behaviours to be screen time (television, computer/game console) and time studying. It was found that the students who performed PA dedicated less time to these sedentary behaviours. These results agree with those of previous investigations that demonstrated lower screen time among physically active people [39,42]. In addition, we established that the male group dedicated more time on the screens while the female students tended to study more, as was suggested by previous studies [43,44]. Although instruments such as tracking devices are often seen as the most valid and trustworthy tools to estimate the levels of PA or sedentary tendencies [45], screen time has also become a standard measure for assessing sedentary behaviour [46], especially among children and young people [47]. Different studies have shown a negative association between screen time (generally television) and health issues for young adults, among which are; an increased risk of excess weight and obesity [48]; neck pain [49]; headaches [50]; difficulty sleeping [51]; depression and anxiety [52]; and reduced general well-being [53]. In our study, the proportion of students who reported feeling low increased significantly among those who were physically inactive.

Others factor that were associated positively with PA, and negatively with sedentary behaviour were perceived health, life satisfaction and happiness [25,54]. 

Our results showed a positive connection between doing PA and self-perception of the students’ physical condition, in that the percentage of students who did PA grew in proportion to the perception of their own physical condition. In addition, those that undertook longer training sessions, also tended to consider themselves in better physical condition. Regarding perception of health, a positive association with PA was only established within the male group. In contrast, a recent study has shown a negative connection between doing PA and perceived health [34]. In this regard, a variable that might influence results is the number of months/year that the subjects have been undertaking PA as a regular routine. A person who has been doing PA over a long period may consider their own health to be good. Whereas a person who is currently doing PA, but who has begun only recently, may consider their health to be poor, and this may be their motivation to begin their new PA routine [34,55]. Indeed, in our study, “health” was one of the most popular reasons for students to justify their PA.

A positive association between taking up healthy dietary habits related and undertaking PA was also demonstrated [39,56]. In our study, this connection appeared between the consumption 4–5 daily meals or snacks and doing PA. Also, the women showed a greater tendency to consume 4–5 daily meals or snacks.

The consumption of alcohol, tobacco and other illegal drugs are considered not to be healthy behaviour. In our study, the smokers did less PA compared with non-smokers. Other studies have obtained similar results, documenting a negative association between the consumption of tobacco and the performance PA [34,39,57]. As for the alcohol, a systematic review showed that participation in sports has a positive association with alcohol consumption. Nevertheless, 80% of the studies found that sports participation was associated with decreased consumption of illegal drugs [57]. In our study, although an association was not established between PA and the consumption of alcohol, a larger proportion of students doing PA was found among those who drank alcohol than among those who did not. 

Another study topic was the influence of interpersonal relationships on levels of PA in young people. Previous studies have shown a positive relation between parents´ performance of PA and a tendency for their children to have physically active lifestyles [58], especially during infancy [59]. In our study we found associations between having physically active fathers, mothers, partners and older siblings and the likelihood of the students themselves doing PA. Although there are studies that suggest that the influence of the each parent is usually greater in children of the same sex [58], or even that the habits of parents, siblings, friends and partners might only be a determinant among women [60], our results showed that, in the case of the male students, PA performance was associated with that of fathers, mothers, friends and partners. In the case of the women, the association was with fathers, mothers, partners and older siblings. These relations might explain, at least in part, how relatives´ levels of PA influence the cardiorespiratory fitness of adolescents [61]. This information reflects the importance of the family in the development of a physically active lifestyle.

In addition to interpersonal factors, it appears that the enjoyment of PE in primary school (especially in women) and secondary school can be a factor that influences the performance of PA during adolescence. Therefore, PE should not only target an increase in moderate-to-vigorous PA during the school years, but also the children´s acquisition of healthy habits related to PA, so that they can be maintained during later stages of life. Our results also showed that the participation in extra-curricular PA organised by schools was associated to PA during university living. Therefore, both PE and educational centres themselves can be seen to be determinant factors for the performance of PA in adolescence and adulthood.

Although the present study has several limitations, the results could be useful in different ways. Firstly, despite the fact that the majority of the sample performed PA, university students stated that they did not perform because little time, in many cases due to lessons and study. In this regard, faculties and school universities should stablish their schedules in coordination with the sports’ services of the universities. In addition, further research about the functioning of the sports’ services of the universities is needed, since they might increase the number of active students with a good offer of activities and timetables. For this, it would be necessary a great economic investment. Secondly, physical education subject seems to have impact in the PA habits of university students. Therefore, the better the training of physical education teachers, the better the PA habits of students. Moreover, in countries as Spain, in many regions elementary and high school students do physical education twice a week, which might not be enough to maximize the benefits of physical education. Finally, taking into account that the PA habits of relatives are positive associated with PA in students, public and private administrations should make more flexible working conditions in order to combine working life with private life.

The present study has some limitations. Being a questionnaire about healthy living habits, it is possible that students answered what they considered to be "politically correct" rather than what they thought, assuming a response bias. On the other hand, some questions formulated in the questionnaire were questions (e.g. perception of health and physical condition); in the future, more objective measures would be included.

## 5. Conclusions

Seven out of every ten university students performed PA, principally to maintain fitness or for health reasons. A lack of time and laziness were seen to be the most common reasons given by those students who chose not to undertake PA, these students, in turn, devoted longer hours to screen time and perceived themselves to be less fit and healthy. 

Furthermore, family members’ levels of PA were reflected by the PA of the university students themselves, as did a sense of satisfaction with PE both in primary and secondary school. These results reveal the importance of the educational system in the adoption and maintenance of healthy lifestyle habits related to the undertaking of PA.

## Figures and Tables

**Table 1:** Characteristics of the sample. Continuous variables expressed as a median (interquartile range); categorical variables expressed as absolute frequency (relative frequency).

Characteristics		N = 608	Male n = 215	Female n = 393
Age in Years		21.0 (19.0–23.0)	21.0 (19.0–23.0)	21.0 (19.0–22.0)
Gender	Male	215 (35.4)	----	----
	Female	393 (64.6)	----	----
Course Year	1º	154 (25.3)	62 (28.8)	92 (23.4)
	2º	118 (19.4)	25 (11.6)	93 (23.7)
	3º	144 (23.7)	56 (26.0)	88 (22.4)
	4º	107 (17.6)	35 (16.3)	72 (18.3)
	5º	62 (10.2)	29 (13.5)	33 (8.4)
	Master	23 (3.8)	8 (3.7)	15 (3.8)
Physical Activity (PA)	Yes	423 (69.6)	159 (74.0)	264 (67.2)
	Week	390 (92.2)	150 (94.3)	240 (90.9)
	Weekend	33 (7.8)	9 (5.7)	24 (9.1)
	No	185 (30.4)	56 (26.0)	129 (32.8)
Minutes/session	Between 10–30	140 (33.2)	26 (12.1)	114 (29.0)
	Between 30–60	125 (29.6)	40 (18.6)	85 (21.6)
	More than 60	157 (37.2)	93 (43.3)	64 (16.3)

**Table 2:** Reasons for undertaking physical activity (PA). Variables expressed as absolute frequency (relative frequency). n = 423. Chi-square in function of gender.

Motive	Sex	Male (n = 159)	Female (n = 264)	χ^2^ p
Fun	Yes	154	236	7.681 0.006
	No	5	28	
To meet friends	Yes	144	204	12.022 0.001
	No	15	60	
To maintain fitness	Yes	156	251	----
	No	3	13	
Enjoyment	Yes	153	235	6.799 0.009
	No	6	29	
To disconnect	Yes	136	231	----
	No	23	33	
Health *	Yes	152	251	----
	No	7	12	
I like to compete	Yes	129	103	71.078 <0.001
	No	30	161	
Personal satisfaction	Yes	153	241	----
	No	6	23	
Competence	Yes	128	179	8.042 0.005
	No	31	85	

* n = 263 women, 1 lost case.

**Table 3:** Categorisation of the reasons to undertake PA.

Motive	Not a Reason	Little Importance	Moderate Importance	Quite Important	Very Important
Fun	33 (7.8)	40 (9.5)	95 (22.5)	121 (28.6)	134 (31.7)
To meet Friends	75 (17.7)	92 (21.7)	92 (21.7)	102 (24.1)	62 (14.7)
To maintain fitness	16 (3.8)	10 (2.4)	41 (9.7)	137 (32.4)	219 (51.8)
Enjoyment	35 (8.3)	56 (13.2)	85 (20.1)	135 (31.9)	112 (26.5)
To disconnect	56 (13.2)	52 (12.3)	93 (22.0)	136 (32.2)	86 (20.3)
Health	20 (4.7)	15 (3.5)	66 (15.6)	140 (33.1)	182 (43.0)
I like to compete	191 (45.2)	94 (22.2)	60 (14.2)	41 (9.7)	37 (8.7)
Personal satisfaction	29 (6.9)	22 (5.2)	90 (21.3)	154 (36.4)	128 (30.3)
Competence	116 (27.4)	107 (25.3)	101 (23.9)	67 (15.8)	32 (7.6)

**Table 4:** Sedentary behaviour by gender. Variables expressed as median (interquartile range).

Sedentary Behaviour				Hours per Day	Mann-Whitney U Test
Study	Mon–Fri		Male	2 (1–4)	0.003
			Female	3 (2–4)	
	Weekend		Male	2 (1–4)	0.001
			Female	3 (1–5)	
	Mon–Sun		Male	2.29 (1.29–3.57)	0.001
			Female	2.71 (1.71–4.14)	
Television	Mon–Fri		Male	1 (0–2)	0.040
			Female	1 (0–2)	
	Weekend		Male	2 (1–3)	----
			Female	2 (1–3)	
	Mon–Sun		Male	1 (0.5–2)	----
			Female	1.29 (0.64–2)	
Computer/Video games	Mon–Fri		Male	2 (1–4)	<0.001
			Female	2 (1–3)	
	Weekend		Male	2.25 (1–4)	<0.001
			Female	2 (1–3)	
	Mon–Sun		Male	2.29 (1.29–3.79)	<0.001
			Female	1.71 (1–2.7)	
Screen time *	Mon–Fri		Male	3 (2–5)	0.027
			Female	3 (2–4)	
	Weekend		Male	4 (3–6)	0.003
			Female	4 (2–6)	
	Mon–Sun		Male	3.46 (2.16–5)	0.043
			Female	3.14 (2.11–4.54)	

* The variable “Screen time” is a combination of television and computer/video games hours.
